# Why Do Students Procrastinate More in Some Courses Than in Others and What Happens Next? Expanding the Multilevel Perspective on Procrastination

**DOI:** 10.3389/fpsyg.2021.786249

**Published:** 2022-02-14

**Authors:** Kristina Kljajic, Benjamin J. I. Schellenberg, Patrick Gaudreau

**Affiliations:** ^1^School of Psychology, Faculty of Social Sciences, University of Ottawa, Ottawa, ON, Canada; ^2^Faculty of Kinesiology and Recreation Management, University of Manitoba, Winnipeg, MB, Canada

**Keywords:** multilevel modeling, well-being, academic achievement, procrastination, motivation

## Abstract

Much is known about the antecedents and outcomes of procrastination when comparing students to one another (i.e., between-person level). However, little is known about the antecedents and outcomes of procrastination when comparing the courses taken by the students during a semester (i.e., within-person level). In this study, we proposed that examining procrastination at both levels of analysis should improve our understanding of the academic experience of students. At both levels, we examined the mediating role of procrastination in the associations between two dimensions of motivation (i.e., autonomous and controlled) and indicators of academic achievement (i.e., grades) and well-being (i.e., positive and negative affect). A sample of 359 university students completed questionnaires measuring their motivation, procrastination, and affect in each of their courses. The official final course grades were obtained at the end of the semester. Multilevel mediation analyses with structural equation modeling were conducted to test our hypotheses. At the between-person level, the indirect effects revealed that higher controlled motivation was significantly associated with worse outcomes (i.e., worse grades and higher negative affect) via higher levels of procrastination. At the within-person level, the indirect effects revealed that lower autonomous motivation was significantly associated with worse outcomes (i.e., worse grades, lower positive affect, and higher negative affect) via higher levels of procrastination. Overall, this study shows that different pathways at each level of analysis may explain how procrastination can be detrimental for the success and well-being of university students.

## Introduction

Learning to manage one’s time is an integral part of university life (e.g., Rothschild-Checroune et al., 2012; Hurst et al., 2013; Abdulghani et al., 2014). Academic tasks (e.g., exams, papers, lab reports, and team projects) often have strict deadlines and students need to study or work on these tasks regularly throughout the semester to avoid feeling overwhelmed at the last minute. Some students struggle to manage their time more than others and thus procrastinate by putting off their academic tasks even though they are aware that their delay will lead to negative consequences (Steel, 2007). The consequences of procrastination are well-documented with meta-analyses showing that it is associated with poorer well-being (van Eerde, 2003; Steel, 2007) and academic achievement (e.g., Richardson et al., 2012; Kim and Seo, 2015). Students who procrastinate more than others thus seem to have more difficulty adjusting to the demands of academic life.

However, students do not behave in the same way across all their courses during a semester. For example, a hypothetical student named Sam might say that they rarely procrastinate in general, but that they frequently procrastinate in their Biology course. Why does Sam procrastinate more in Biology than in their other courses? What are the consequences of Sam’s frequent procrastination in Biology? A recently proposed multilevel perspective on procrastination across courses has shown that within-person course procrastination is negatively related to within-person final course grade (Kljajic and Gaudreau, 2018). This means that Sam would be expected to have a lower grade in Biology than in their other courses. As a next step in this new research venue, we propose to expand the nomological network of procrastination by examining its antecedents and outcomes. More precisely, we tested the mediating role of procrastination in the relations between two dimensions of motivation and indicators of achievement and emotional well-being both across students (i.e., between-person level) and across the courses taken by each student during a semester (i.e., within-person level).

### Two Dimensions of Motivation, Academic Achievement, and Well-Being

Students have various reasons for being engaged in their academic courses. According to Self-Determination Theory (SDT; Deci and Ryan, 2000; Ryan and Deci, 2020), these reasons can be described as regulatory styles and classified into two dimensions of motivation. The first dimension, called *autonomous motivation*, includes regulatory styles that reflect a deliberate choice to do an activity. Students with high autonomous motivation are engaged in their academic tasks because they are perceived as inherently interesting and enjoyable (i.e., intrinsic regulation), as closely aligned with their foundational values and interests (i.e., integrated regulation), or as personally valuable (i.e., identified regulation). The second dimension of motivation, called *controlled motivation*, includes regulatory styles that reflect internal or external pressure to do an activity. Students with high controlled motivation are engaged in their academic tasks because they want to avoid feelings of shame or guilt due to failure or want to experience better self-esteem due to success (i.e., introjected regulation) or because they want to receive a reward or avoid a punishment (i.e., external regulation). One of the main hypotheses of SDT is that more autonomous regulatory styles should be associated with more engagement, learning, and well-being in the academic domain (Ryan and Deci, 2020). As such, autonomous and controlled motivation are expected to be related differently to outcomes, with autonomous motivation being related to better academic outcomes.

Motivation is often considered as an important ingredient for academic success (Guay et al., 2008). Students who do their academic tasks for autonomous reasons would be most likely to be driven to produce high-quality work and, as a result, achieve very good grades. Several meta-analytical findings have shown that indicators of autonomous motivation (i.e., intrinsic and/or identified regulation) are positively associated with achievement in the academic domain (Richardson et al., 2012; Cerasoli et al., 2014; Taylor et al., 2014; Howard et al., 2021). Likewise, recent studies with university students have generally found that autonomous motivation is significantly and positively correlated to academic achievement (e.g., Bonneville-Roussy et al., 2017; Gareau and Gaudreau, 2017; Yu et al., 2018; Orsini et al., 2019). By contrast, students who do their academic tasks for controlled reasons would most likely do just enough to finish their work without necessarily spending more time on improving its quality. One small-scale meta-analysis with studies using only the Academic Motivation Scale (Vallerand et al., 1992) has shown that indicators of controlled motivation (i.e., introjected and external regulation) are negatively associated with academic achievement (Taylor et al., 2014). However, another broader and more recent meta-analysis has instead found no significant association between indicators of controlled motivation and academic achievement (Howard et al., 2021). Similarly, recent studies with university students have generally found a negative, albeit mostly non-significant correlation between controlled motivation and academic achievement (e.g., Bonneville-Roussy et al., 2017; Jeno et al., 2018; Gareau et al., 2019; Aydin and Michou, 2020). Overall, these findings indicate that autonomous and controlled motivation play a differential role on academic achievement. Although the evidence of the negative role played by controlled motivation is somewhat inconclusive, having more autonomous motivation is clearly a relevant ingredient for greater academic success.

Motivation is also considered as a key variable that can enhance the emotional well-being of students (Ryan and Deci, 2009). Students with high autonomous motivation freely choose to do their academic tasks and, for them, those tasks are perceived as interesting, valuable or personally important. Therefore, such students are more likely to experience better emotional well-being. Meta-analytical findings support this proposition by showing that indicators of autonomous motivation (i.e., intrinsic and identified regulation) are related to higher positive affect and lower negative affect in the academic setting (Howard et al., 2021). Also, when affect is measured in a dispositional/general way or during a medium time-frame (e.g., over a few weeks), autonomous motivation is correlated positively to positive affect and negatively to negative affect (e.g., Litalien et al., 2013, 2015; Waaler et al., 2013; Garn et al., 2019). Conversely, students with high controlled motivation feel an obligation to do academic tasks due to internal or external pressures and, as a result, they are more likely to experience conflicted emotions. A recent meta-analysis showed that indicators of controlled motivation (i.e., introjected and external regulation) are positively related to negative affect; however, conflicting evidence is found for positive affect (Howard et al., 2021). When affect is measured in a dispositional/general way or during a medium time-frame, negative affect is usually significantly and positively correlated to controlled motivation, whereas the correlation between positive affect and controlled motivation tends to be negative but mostly non-significant (e.g., Litalien et al., 2015; Garn et al., 2019; Sanjuán and Ávila, 2019). Taken together, these results suggest that autonomous motivation is related to better emotional well-being (i.e., higher positive affect and lower negative affect), whereas controlled motivation is related to worse emotional well-being to a certain extent (i.e., higher negative affect).

**FIGURE 1:**
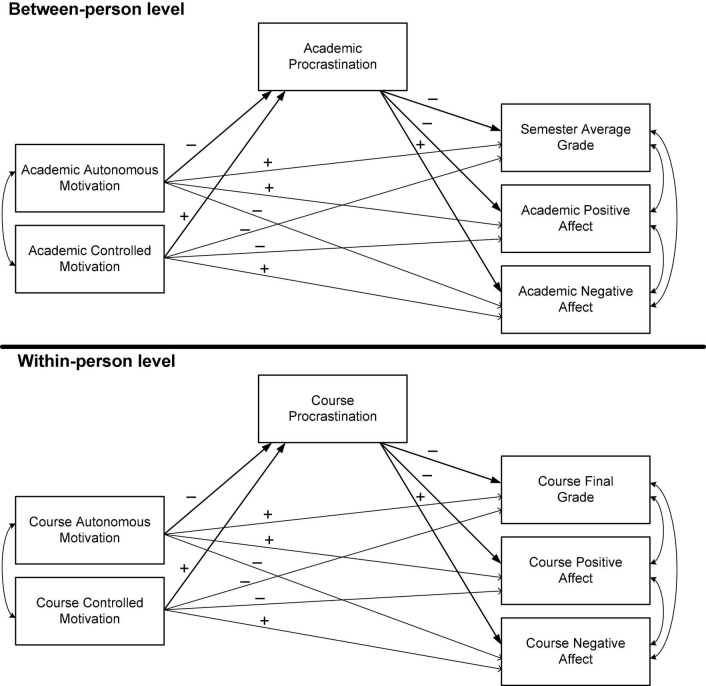
Hypothesized multilevel mediation model.

In order to experience success and well-being, students benefit not only from the right type of motivation, but also from an ability to prioritize the completion of academic tasks over other appealing options (e.g., spending time with friends, watching a movie). When students delay starting or completing an academic task even though they anticipate the negative consequences due to their delay, they are said to be procrastinating (Steel, 2007). Students who procrastinate tend to produce low-quality work and experience more negative affect, such as anger, anxiety, shame, sadness, and dissatisfaction (Patrzek et al., 2012; Grunschel et al., 2013). As shown in Figure 1, we propose that procrastination could act as a mediator that can explain how two dimensions of motivation (i.e., autonomous and controlled) relate to academic outcomes (i.e., achievement and affect).

### The Mediating Role of Procrastination

Motivation is recognized as playing an important role in the capacity to self-regulate (Molden et al., 2016). On the one hand, students with high autonomous motivation – who perceive their courses as engaging or valuable – would be less likely to constantly and irrationally delay the completion of their academic tasks. Accordingly, studies have found, for the most part, that autonomous motivation is significantly and negatively correlated to procrastination in the academic domain (e.g., Vansteenkiste et al., 2009; Katz et al., 2014; Mouratidis et al., 2018). Indicators of autonomous motivation (i.e., intrinsic and identified regulation) were also mostly negatively correlated to procrastination (Senécal et al., 1995; Lee, 2005; Seo, 2013). On the other hand, students with high controlled motivation – who put pressure on themselves or perceive an external pressure to work on their academic tasks – would be more likely to frequently postpone the completion of their academic tasks to another day. Indeed, most studies in the academic domain have shown a significant and positive correlation between controlled motivation and procrastination (e.g., Vansteenkiste et al., 2009; Mih, 2013; Mouratidis et al., 2018). Furthermore, indicators of controlled motivation (i.e., introjected and/or external regulation) tend to be positively – although not necessarily significantly – correlated to procrastination (Senécal et al., 1995; Lee, 2005; Seo, 2013). Overall, despite the limited number of studies conducted on the two dimensions of motivation and procrastination in the academic setting, the results seem to be fairly consistent: Autonomous and controlled motivation are generally related to less and more procrastination, respectively.

Procrastination is expected to have a negative influence on academic achievement. Many students who procrastinate eventually experience the time pressure to complete an academic task before a deadline and the accumulation of multiple academic tasks to complete at the same time (Patrzek et al., 2012; Grunschel et al., 2013). As a result, these students often do not have enough time to produce high-quality work and get excellent grades. Meta-analytical findings have shown that students who procrastinate more tend to obtain lower grades than students who procrastinate less (van Eerde, 2003; Steel, 2007; Richardson et al., 2012; Kim and Seo, 2015). Even in the courses in which university students procrastinate more than their own average, they tend to obtain lower grades compared to their own average (Kljajic and Gaudreau, 2018). Hence, studies have generally shown that procrastination is negatively related to academic achievement.

Procrastination is also expected to be related to worse emotional well-being. When students procrastinate, they might experience temporary relief by avoiding an academic task that is considered aversive. However, procrastination tends to increase self-generated stress and students often end up feeling worse after they procrastinate (Sirois and Pychyl, 2013). A small-scale meta-analysis with samples of community participants and students revealed that procrastination was significantly negatively associated with positive affect (*r* = −0.29) and significantly positively associated with negative affect (*r* = 0.31; Sirois et al., 2019). Likewise, many studies conducted with university students have shown that procrastination is associated with lower positive affect and greater negative affect (e.g., Turban et al., 2013; Blouin-Hudon and Pychyl, 2015; Balkis and Duru, 2016; Choy and Cheung, 2018; Gautam et al., 2019). Overall, these findings indicate that procrastination is related to worse emotional well-being (i.e., lower positive affect and higher negative affect).

### The Present Study

The multilevel perspective proposed by Kljajic and Gaudreau (2018) to measure procrastination and grades across courses was used as a foundation to develop the present study. In their study, Kljajic and Gaudreau (2018) examined the relation between procrastination and grades across students (i.e., between-person level) and across the courses taken by each student during the semester (i.e., within-person level). First, they found that a large percentage of the variance (i.e., 39%) in procrastination and grades was due to the fluctuations across courses (i.e., within-person level), which warranted the usage of a multilevel model. Second, they found a significant and negative association between procrastination and grades at both levels of analysis. However, the results were interpreted differently at each level of analysis. At the between-person level, they found that students who procrastinated *more than others* tended to obtain a lower average grade than others. At the within-person level, they found that in the courses in which students procrastinated *more than their own average*, they tended to obtain a lower final course grade than their own average. Taken together, these results revealed the value of investigating procrastination across the courses taken by university students during a semester.

The present study also measured the relation between procrastination and grades at two levels of analysis. However, we also built upon the study of Kljajic and Gaudreau (2018) in two ways: (a) we proposed two motivational antecedents of procrastination, and (b) we added two indicators of emotional well-being as outcomes of procrastination. More specifically, we investigated the mediating role of procrastination in explaining how autonomous and controlled motivation relate to three academic outcomes (i.e., grades, positive affect, and negative affect) at the between-person and within-person levels of analysis. We hypothesized that the findings would be comparable at the two levels (see Figure 1). However, the results would hold a distinct conceptual meaning at each level.

#### Between-Person Interpretation (Comparing Students to One Another)

We hypothesized that students with lower autonomous motivation and higher controlled motivation would tend to procrastinate more. In turn, we hypothesized that students who procrastinate more would tend to have a lower average grade and positive affect and a higher negative affect.

#### Within-Person Interpretation (Comparing Courses to One Another Within Each Student)

In the courses in which students have lower autonomous motivation and higher controlled motivation, we hypothesized that they would tend to procrastinate more. In turn, in the courses in which students procrastinate more, we hypothesized that they would tend to have lower course grades and course positive affect, and higher course negative affect.

## Materials and Methods

### Participants

Participants were recruited in the student pool of the integrated system for participation in research at the University of Ottawa, in Canada. These students were recruited in their first-year introductory psychology or linguistics courses or in their second-year research methods in communication course. Participants in our study were 359 undergraduate students (71.3% female and 28.7% male). Their age ranged from 16 to 46 years (*M* = 19.27, *SD* = 3.24) and they were in their first (68.2%), second (18.1%), third (9.7%), or fourth (3.9%) year of study. Participants were enrolled in health sciences (30.4%), social sciences (29.2%), science (22.6%), arts (7%), engineering (2.8%), management (1.7%), or in multiple faculties (6.1%). They reported that they were taking two (0.8%), three (1.9%), four (22.3%), five (71%), or six (3.9%) courses during the semester. Participants described their ethnic background as European-Canadian/White (46.4%), Asian (22.3%), Arabic (10.9%), African-Canadian/Black (6.1%), Hispanic/Latino (1.7%), Aboriginal/Native (0.6%), or other (12%). They were living with their parents (42.6%), in a residence (29.7%), or in an apartment (27.7%) and they received a full scholarship (3.9%), a partial scholarship (57%), or no scholarship (39.1%) to cover their tuition fees. Some students (41.2%) had to work during the semester and they worked on average 14.86 h per week. This study was approved by the Research Ethics Board of the university.

### Procedure

During the fall semester 2019, university students were recruited to participate in a study on their academic experience in each of their courses. The recruitment was conducted online via an integrated system for participation in research. We opened the survey a month after the beginning of the semester to ensure that the participants could reflect on their general experience in each of their courses. All participants completed the consent form for the study. Participants completed demographic and socio-economic questions before they reported how many courses they were taking in the current semester using a drop-down menu including options from one to six courses. Participants were then redirected to the course-related part of the survey that matched their answer on the drop-down menu. For example, if a participant indicated that they had five courses, they were redirected to the webpage “Questions about your five courses” in which they were asked to complete the same questionnaires for each course. For each course, participants were asked to report the course code (e.g., PSY-1101-C) and the name of the professor before they completed questionnaires referring to that course. After the questionnaires for the last course were completed, participants were thanked for their participation in the study and they received one point toward their introductory psychology or linguistics course or their research methods in communication course as compensation.

### Measures

#### Course Autonomous and Controlled Motivation (Independent Variables)

A 10-item questionnaire was used to measure motivation according to SDT (Gareau and Gaudreau, 2017). Participants indicated the extent to which each item corresponded to a reason why they were enrolled in the referred course on a scale from 1 (*not at all for this reason*) to 7 (*totally for this reason*). The items measuring intrinsic regulation (e.g., “Because I truly love it”; two items), integrated regulation (e.g., “It is part of who I am as a person”; two items), and identified regulation (e.g., “In order to pursue goals that are important to me”; two items) were averaged to create a score of autonomous motivation (within-person α = 0.83; between-person α = 0.91). The items measuring introjected regulation (e.g., “Otherwise, I would feel guilty”; two items) and external regulation (e.g., “Somebody is putting pressure on me”; two items) were averaged to create a score of controlled motivation (within-person α = 0.74; between-person α = 0.94).

#### Course Procrastination (Mediating Variable)

A 3-item questionnaire was used to measure procrastination (Kljajic and Gaudreau, 2018) based on items from past questionnaires (Solomon and Rothblum, 1984; Steel, 2010). For each course, participants were presented with a definition of procrastination (“Procrastination refers to when we delay starting or completing some task even though we are aware that negative consequences will probably follow the delay”) and were then asked to think about their procrastination on academic tasks (e.g., studying for an exam or writing a term paper) outside of class. They then rated the degree to which they agreed with each item for the referred course on a scale from 1 (*Not at all agree*) to 5 (*Totally agree*). The reliability of this questionnaire was good in this study (within-person α = 0.84; between-person α = 0.93).

#### Course Final Grade (Dependent Variable)

For the purposes of this study, participants were given the option to grant access to their official final grades in each of their courses at the end of the semester via the Registrar’s Office. A total of 240 participants (67% of the sample) agreed to grant access to their final grades in all their courses. The final grades were based on the official 11-point grading system used by the university: 0 = *F* (0–39%), 1 = *E* (40–49%), 2 = *D* (50–54%), 3 = *D* + (55–59%), 4 = *C* (60–64%), 5 = *C* + (65–69%), 6 = *B* (70–74%), 7 = *B* + (75–79%), 8 = *A* − (80–84%), 9 = *A* (85–89%), and 10 = *A* + (90–100%).

#### Course Positive and Negative Affect (Dependent Variables)

A 9-item questionnaire was used to measure positive and negative affect (Emmons, 1992). Participants indicated the extent to which they felt a certain way over the past 2 weeks in the referred course on a scale from 1 (*Not at all*) to 7 (*Totally*). Four adjectives measured positive affect (e.g., happy and joyful) and five adjectives measured negative affect (e.g., frustrated and worried/anxious). In this study, the reliability of this questionnaire was excellent for positive affect (within-person α = 0.94; between-person α = 0.95) and very good for negative affect (within-person α = 0.88; between-person α = 0.91).

### Plan of Analyses

The analyses were conducted in M*plus* version 8 (Muthén and Muthén, 1998-2017). We used Multilevel Structural Equation Modeling (MSEM; see Preacher et al., 2010) to test our hypothesized mediation model at each level of analysis. In MSEM, the within-person variables are implicitly group-mean centered (Preacher et al., 2010). The full information maximum likelihood (FIML) was used to handle missing data and the robust maximum likelihood estimator (MLR) was used to obtain robust standard errors and chi-square test statistics correcting for non-normality. All paths were treated as fixed effects, because random effects are computationally demanding for such a complex model and would lead to errors in model estimation.^1^ Multiple fit indices were used, namely the chi-square (χ^2^), CFI, TLI, RMSEA, and SRMR (the last one includes a value at each level of analysis). Nested models were compared with a scaled chi-square difference test (Satorra and Bentler, 2010) using an online calculator^2^.

## Results

### Preliminary Analyses

When we examined our initial sample of 378 university students, we found that 12 participants did not complete any of the relevant questionnaires for this study and we thus removed them for the analyses. We then examined whether the sample included potential outliers. At the within-person level, we calculated the within-person correlations between all the variables of interest for each participant. Each participant could have up to 15 within-person correlations, which were then transformed into *z*-scores. At the between-person level, the average scores of each variable were also transformed into *z*-scores. Participants were considered to be potential outliers at each level of analysis if the two following conditions were met: (a) they had a *z*-score above 3 or below -3 and (b) they were clearly apart from the rest of the sample based on a visual inspection of the distribution. Furthermore, participants could be considered as potential multivariate outliers at the between-person level if their computed score of Mahalanobis distance was higher than the critical value [χ^2^(6) = 22.46, *p* < 0.001]. Based on these screening methods, we found no outliers at the within-person level and seven outliers at the between-person level. We conducted the analyses with the outliers (*n* = 366) and without the outliers (*n* = 359) and we found that some parameters changed considerably once the outliers were removed. Therefore, we decided to exclude the seven outliers for the analyses and our final sample included 359 university students.

Descriptive statistics and bivariate correlations can be found in Table 1. Of particular interest, we found substantial within-person variability for all the variables in this study (i.e., between 34 and 76%). In other words, all the variables fluctuated considerably across the courses that the students were taking during the semester, which indicated the relevance of using a multilevel framework to test our hypotheses.

**TABLE 1:** Descriptive statistics and bivariate correlations at each level of analysis.

	*M ^a^*	*SD ^a^*	*SD ^b^*	1 – ICC	1	2	3	4	5	6
(1) Autonomous motivation	3.10	0.90	1.06	0.58	−	0.06*	−0.24*	0.11*	0.59*	−0.26*
(2) Controlled motivation	1.59	0.81	0.59	0.34	0.34*	−	0.04	−0.09*	−0.10*	0.16*
(3) Procrastination	2.56	0.86	0.77	0.44	0.02	0.24*	−	−0.24*	−0.34*	0.35*
(4) Grades	6.55	1.86	1.64	0.44	0.08	−0.21*	−0.38*	−	0.30*	−0.36*
(5) Positive affect	3.40	0.74	1.33	0.76	0.60*	0.22*	0.00	0.03	−	−0.52*
(6) Negative affect	2.60	0.77	1.10	0.67	0.26*	0.51*	0.33*	−0.21*	0.26*	−

*p < 0.05. Within-person N = 1668 courses. Between-person N = 359 students. Between-person correlations are below the diagonal and within-person correlations are above the diagonal. ICC: intra-class correlation. 1 – ICC: the amount of within-person variance. ^a^Between-person level. ^b^Within-person level.

We conducted a step-by-step model building approach to determine the model that would provide the best fit to the data while also being the most parsimonious (see Table 2 for the fit indices of each model). In Model 1, procrastination was assumed to be a full mediator of the relations between the two dimensions of motivation and academic outcomes. None of the direct effects were thus estimated at both levels of analysis. All the fit indices of this first model were poor and unacceptable. In Model 2, procrastination was assumed to be a full mediator at the between-person level, but a partial mediator at the within-person level. That is, all the direct effects were added at the within-person level only. The scaled chi-square difference test revealed that Model 2 provided a better fit to the data compared to Model 1. However, the fit indices of the second model remained unsatisfactory for the most part. All the within-person direct effects were significant and were thus kept in the next model. In Model 3, procrastination was assumed to be a partial mediator at both levels of analysis. In other words, all the direct effects were also added at the between-person level. The scaled chi-square difference test showed that Model 3 provided a better fit than Model 2. However, the third model was fully saturated (i.e., zero degree of freedom). Two between-person direct effects were found to be non-significant, namely the relation between controlled motivation and positive affect and the relation between autonomous motivation and negative affect. These two direct effects were removed in the fourth model. The scaled chi-square difference test indicated that Model 4 did not significantly differ from Model 3. Nevertheless, the fit indices of Model 4 were excellent and this model was more parsimonious than Model 3. Therefore, Model 4 was chosen as the final model.

**TABLE 2:** Fit indices and comparisons of the tested models.

	χ^2^	*df*	CFI	TLI	RMSEA	SRMR_*within*_	SRMR_*between*_	Contrast	Δ*df*	Δχ^2^
Model 1	663.719*	12	0.514	−0.216	0.180	0.123	0.178			
Model 2	139.724*	6	0.900	0.501	0.116	0.009	0.179	1 vs. 2	6	470.846*
Model 3	0	0	1.000	1.000	0.000	0.000	0.000	2 vs. 3	6	139.724*
Model 4	3.255	2	0.999	0.986	0.019	0.002	0.030	3 vs. 4	2	3.255

*p < 0.05. Model 1: none of the direct effects was estimated at both levels of analysis. Model 2: direct effects were estimated at the within-person level only. Model 3: direct effects were estimated at both levels of analysis. Model 4: direct effects were estimated at both levels of analysis except for two non-significant direct effects at the between-person level.

### Main Analyses

**FIGURE 2:**
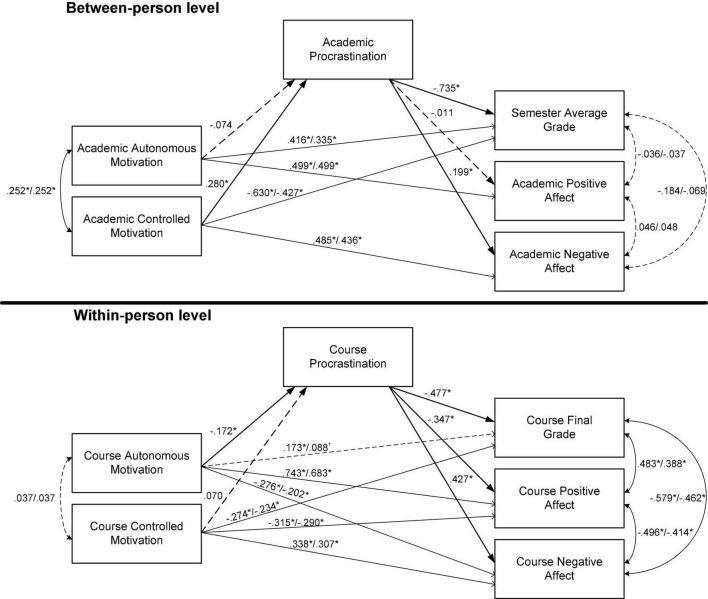
Unstandardized parameter estimates at each level of analysis. *p < 0.05, ^†^*p* < 0.10. Full lines represent significant relations; dashed lines represent non-significant or marginally significant relations. The value at the left of the slash is the estimate in the total effect model; the value at the right of the slash is the estimate in the direct effect model.

The unstandardized parameter estimates of the final model at both levels of analysis are illustrated in Figure 2. The total, direct, and indirect effects are shown in Table 3.

**TABLE 3:** Unstandardized estimates and confidence intervals of the total, direct, and indirect effects at each level of analysis.

	Total effect		Direct effect		Indirect effect	
	*B*	95% CI	*B*	95% CI	*B*	95% CI
**Between-person level**						
Autonomous motivation to grades	0.416*	[0.057, 0.776]	0.335*	[0.022, 0.649]	0.055	[−0.045, 0.155]
Controlled motivation to grades	−0.630*	[−0.982, −0.279]	−0.427*	[−0.780, −0.074]	−0.206*	[−0.325, −0.086]
Autonomous motivation to positive affect	0.499*	[0.395, 0.602]	0.499*	[0.395, 0.604]	0.001	[−0.008, 0.010]
Controlled motivation to positive affect	0^a^		0^a^		–0.003	[−0.035, 0.029]
Autonomous motivation to negative affect	0^a^		0^a^		–0.015	[−0.042, 0.012]
Controlled motivation to negative affect	0.485*	[0.365, 0.605]	0.436*	[0.310, 0.561]	0.056*	[0.017, 0.094]
**Within-person level**						
Autonomous motivation to grades	0.173*	[0.077, 0.269]	0.088^†^	[−0.004, 0.179]	0.082*	[0.049, 0.115]
Controlled motivation to grades	−0.274*	[−0.474, −0.073]	−0.234*	[−0.429, −0.039]	–0.034	[−0.077, 0.010]
Autonomous motivation to positive affect	0.743*	[0.678, 0.807]	0.683*	[0.618, 0.747]	0.060*	[0.037, 0.082]
Controlled motivation to positive affect	−0.315*	[−0.405, −0.225]	−0.290*	[−0.380, −0.199]	–0.024	[−0.055, 0.006]
Autonomous motivation to negative affect	−0.276*	[−0.340, −0.213]	−0.202*	[−0.262, −0.142]	−0.073*	[−0.100, −0.047]
Controlled motivation to negative affect	0.338*	[0.212, 0.465]	0.307*	[0.193, 0.422]	0.030	[−0.009, 0.069]

*p < 0.05, ^†^p < 0.10. All indirect effects were tested via procrastination. ^a^The total/direct effect was fixed at zero.

#### Between-Person Findings

Autonomous motivation was associated with higher grades and positive affect. By contrast, controlled motivation was associated with lower grades and higher negative affect. Procrastination did not significantly mediate the relations between autonomous motivation and any of the outcomes. However, procrastination was a significant mediator of the relations between controlled motivation and two of the outcomes (i.e., grades and negative affect). Students who experienced more controlled motivation than others were more likely to procrastinate more than others. In turn, students who procrastinated more than their counterparts were more likely to get worse grades and experience more negative affect than their counterparts.

#### Within-Person Findings

Autonomous motivation was associated with marginally higher grades, higher positive affect, and lower negative affect. Conversely, controlled motivation was associated with lower grades, lower positive affect, and higher negative affect. Procrastination did not significantly mediate the relations between controlled motivation and any of the outcomes. However, procrastination was a significant mediator of the relations between autonomous motivation and all the outcomes. In the courses in which students experienced less autonomous motivation than their own average, they were more likely to procrastinate more than their own average. In turn, in the courses in which students procrastinated more than their own average, they were more likely to get worse grades and experience less positive affect and more negative affect than their own average.

## Discussion

The current study was designed to take into consideration that procrastination fluctuates not only from one university student to another (i.e., between-person level), but also across the courses that each student is taking during a semester (i.e., within-person level). With the goal of also investigating its nomological network, we proposed that procrastination could mediate the influence of two dimensions of motivation (i.e., autonomous and controlled) on academic achievement (i.e., grades) and well-being (i.e., positive and negative affect) at both levels of analysis. Our main finding was that the antecedent of procrastination differed across levels of analysis. On the one hand, procrastination was negatively associated with *between-person* differences in achievement and well-being because some students experienced more controlled motivation than others. On the other hand, procrastination was negatively associated with *within-person* differences in achievement and well-being because students experienced less autonomous motivation in some courses compared to others. Overall, our results showcase that rich insights can be gained by looking at procrastination and its nomological network through the lens of a multilevel perspective.

### Expanding the Multilevel Perspective on Procrastination

The most consistent finding at both levels of analysis was that procrastination was negatively associated with academic achievement and well-being. More precisely, higher procrastination was related to lower grades and more negative affect at both levels, while also being related to less positive affect at the within-person level. These findings are consistent with the recent multilevel study of Kljajic and Gaudreau (2018) which has shown a negative relation between procrastination and grades across students (i.e., between-person level) and across the courses taken by each student (i.e., within-person level). Our results also corroborate meta-analytical findings at the between-person level showing that procrastination relates to lower academic achievement, more psychological maladjustment (van Eerde, 2003; Steel, 2007), and more negative affect (Sirois et al., 2019). Although the outcomes of procrastination are comparable at both levels of analysis, the conceptual meaning and implications of our results differ at each level. If we refer to the fictional example presented in the introduction, Sam – who has a low score of general procrastination – would probably have a better average grade and experience less negative affect than their counterparts who procrastinate more. Yet, because Sam procrastinates more in Biology compared to their other courses, we would expect Sam to have a lower grade and experience less positive affect and more negative affect in Biology compared to their other courses. Overall, these results highlight that although students like Sam probably do not struggle as much as their counterparts with their procrastination, they are still at risk of experiencing negative outcomes in the courses in which they procrastinate more.

Based on these findings, we might wonder how to help students who struggle with their procrastination in different ways. First, what are some ideas that could potentially help students who – unlike Sam – tend to procrastinate a lot in general? At the between-person level, we found that students with high controlled motivation tend to procrastinate more, which is consistent with results found in past studies (e.g., Vansteenkiste et al., 2009; Mih, 2013; Mouratidis et al., 2018). Thus, if a student procrastinates more than students procrastinate on average, our finding suggests that decreasing controlled motivation (rather than increasing autonomous motivation) would potentially be a good way to reduce their procrastination. It is possible that students who experience high controlled motivation in university feel an external pressure from significant others to be enrolled in a program of study or at an academic institution that is not closely aligned with their own values, interests, and priorities. As such, these students may have enrolled in a program or at an institution that would not have been their personal choice. Furthermore, they may believe that they do not have the required skills to succeed in this program or they might not feel connected to their colleagues in the program. Understanding how basic psychological needs according to SDT (i.e., autonomy, competence, and relatedness; e.g., Ryan and Deci, 2000) are associated with procrastination via controlled motivation could provide some important insights to eventually develop interventions that could help students who generally procrastinate across all of their courses.

Second, what are some ideas that could potentially help students who – like Sam – procrastinate more in some specific courses? At the within-person level, we found that students tend to procrastinate more than their own average in the courses in which they have lower autonomous motivation. Therefore, if a student procrastinates more in a course compared to their own average, our finding suggests that increasing autonomous motivation (rather than decreasing controlled motivation) could be a good way to reduce their procrastination in that course. An avenue that may increase autonomous motivation in the classroom would be for professors to use teaching strategies that support autonomy (Reeve and Jang, 2006), such as giving options to students whenever possible (e.g., evaluation methods, topic of the term paper), explaining why a given academic project that may seem uninteresting is relevant and important for their career, and encouraging an active learning process by creating small discussion groups and asking the students to discuss and summarize a course topic (e.g., Kusurkar et al., 2011). Alternatively, students could perhaps benefit from personal strategies to increase their autonomous motivation outside of the classroom. For example, in a course in which a student has low autonomous motivation, they could write a short paragraph explaining why they believe the topic of a book chapter is relevant to their life or useful for them before they start studying it (e.g., Hulleman et al., 2010; Canning et al., 2018). Such strategies would be especially important in compulsory courses that students are required to take to graduate, compared to elective courses that are selected by choice. All of these strategies could potentially increase the autonomous motivation of a student in a given course. However, we do not know yet whether such strategies could also reduce the procrastination of a student in that course. In future studies, randomized field trials could be designed to assess the extent to which course autonomy-supportive strategies – both inside and outside the classroom – could increase course autonomous motivation and, in turn, reduce course procrastination.

On a final note, we might wonder if students with high levels of average academic procrastination may benefit from focusing on reducing their procrastination in a specific course. In some cases, reducing the general academic procrastination by reducing the controlled motivation of these students may not be a realistic option. For example, if they have already invested a significant amount of resources in their education, some students may feel that they do not have the luxury to change their program or academic institution. Could these students still benefit from attempting to reduce their procrastination in a given course? On the one hand, intervening at such a contextual level may not be enough to reduce the general academic procrastination of a student who already procrastinates a lot in school. On the other hand, having at least one course in which a student experiences more autonomous motivation and less procrastination may spillover to positively influence their experiences in other courses or potentially even encourage the student to decrease their procrastination in general. After all, courses are not completely independent of each other; they coexist within a same timeframe (i.e., academic semester). Our findings offer novel insights to inspire future randomized field trials to determine whether attempting to decrease procrastination in a course can influence other courses or the general academic procrastination of a student.

### Limitations and Future Directions

An unexpected finding in this research was that autonomous motivation was not significantly correlated to procrastination at the between-person level (*r* = 0.02). This finding stands apart from a few past studies which have found a moderate negative correlation between autonomous motivation and procrastination (*r* = −0.40 to −0.50; Vansteenkiste et al., 2009; Katz et al., 2014; Mouratidis et al., 2018; for an exception, see Mih, 2013). This difference in the results can potentially be due to the way that the variables have been measured. In past studies, the variables have been measured with a single questionnaire assessing the general experience of students in a given task such as studying (Vansteenkiste et al., 2009; Study 1, Mouratidis et al., 2018) or doing homework (Katz et al., 2014) or the experience in a given course (Vansteenkiste et al., 2009; Study 2). In our study, motivation and procrastination at the between-person level are instead a combination of the experiences across all courses taken by each student. When students complete a general questionnaire, it is unlikely that they use a mental algorithm to calculate their average experience across all their courses. Instead, their assessment might even be partially biased by a course in which they have a strong positive or negative experience. In future research, we would thus encourage researchers to measure the variables at the between-person level both with a general questionnaire and a summation of experiences across courses to determine whether methodology has a significant influence on the results.

Another finding that would merit further attention is the association between controlled motivation and academic achievement. In our study, we found a significant small-to-moderate negative correlation between these two variables at the between-person level. Although this finding was expected, it does not explain why past empirical evidence on the association between controlled motivation and academic achievement has been inconclusive. To allow us to partially speculate on this topic, we have examined two past studies that have used the same questionnaire of academic motivation and the same measure of objective academic achievement that we used in our study. Both previous studies have shown a non-significant correlation between controlled motivation and academic achievement (Gareau and Gaudreau, 2017; Gareau et al., 2019). One of the possible reasons why our finding differs from the results of these two studies might be because our instructions in the motivation questionnaire are not the same. In our study, participants were asked why they are enrolled in each of their courses whereas, in the two previous studies, participants were asked why they pursue academic activities. When thinking about academic activities in general, students might not only think about their experience in their courses, but also about their general experience on campus (e.g., involvement in student associations, recreational, or competitive sports). Alternatively, they may think only about their courses, but their way of “averaging” their motivation might not be the same as when we compute their motivation score across all their courses. Future research could randomize participants in different groups that use the same motivation questionnaire, but with slightly different instructions, to determine whether this experimental manipulation has an influence on students’ responses and ultimately, on the correlation between controlled motivation and academic achievement.

In our study, we proposed that procrastination would influence affect. Accordingly, participants were asked to report their course procrastination in general during the semester and their course affect only during the last 2 weeks. Our reasoning was that participants were likely to think about procrastination episodes that occurred before the assessment of their affect. However, the procrastination episodes could have occurred at the same time and even after their reported affect. For example, an academic task that was perceived as being unclear and uninteresting could have increased the negative affect of a student (e.g., unhappy and frustrated) and, in turn, that student would have been more likely to procrastinate (see Sirois and Pychyl, 2013). Although future studies are needed, untangling the complex bidirectional relations between procrastination and affect may prove to be especially difficult because students experience many fluctuations in procrastination and affect within a short time span (see Pychyl et al., 2000). Nonetheless, our multilevel perspective offers a useful framework to inspire future studies using experience sampling methods. Participants could complete short questionnaires measuring affect and procrastination multiple times during the day (level 1) for several consecutive days (level 2) in the lives of each student (level 3). We could then properly estimate whether previous affect influences subsequent procrastination, whether previous procrastination influences subsequent affect, or both.

We found a noteworthy difference in direction of the correlation between positive and negative affect when comparing the two levels of analysis. At first glance, these findings may seem contradictory. However, this is not a novel finding (e.g., Dunkley et al., 2014; Rush and Hofer, 2014) and previous theories and research have already proposed explanations for this phenomenon (Russell and Carroll, 1999). On the one hand, the correlation is *negative* at the within-person level. In the courses in which students experience more positive affect (e.g., happy and joyful) than their own average, they also tend to experience less negative affect (e.g., frustrated and worried/anxious). We could thus suggest that university students are unlikely to simultaneously experience strong positive and negative affect in the same course within a moderate time frame of 2 weeks. On the other hand, the correlation is *positive* at the between-person level. Students who experience more academic positive affect also tend to experience more academic negative affect than other students. In their broader academic lives (across all of their courses), students are more likely to experience a wider range of affect-inducing events likely to produce both positive and negative emotions (see Folkman and Moskowitz, 2000). Overall, these findings illustrate why a multilevel perspective can be useful to uncover associations likely to differ across levels of analysis.

Most variables in this study were measured at the same time, except for grades which were obtained at the end of the semester. Despite the limitations of a cross-sectional design, this type of design is wise to use as a time-efficient and cost-effective way of answering questions in a new research program (Spector, 2019). In our study, we first needed to establish whether the variables of interest were significantly related – especially at the within-person level – before proposing more complex designs (e.g., longitudinal and experimental). Although we have proposed a theoretically-sound mediational model and we want to encourage researchers to replicate this study, we also recognize that focusing mostly on cross-sectional associations is limitative and does not allow us to infer causality. Future studies could thus come up with more robust designs that could rule out alternative explanations (e.g., adding control variables such as goal setting) or even potentially infer causality. For example, researchers could teach students some strategies to increase their autonomous motivation and then examine whether in the courses in which students use these strategies more often at the beginning of the semester, they are less likely to procrastinate later in the semester.

One of the main goals of this study was to examine the antecedents and outcomes of procrastination, which is why procrastination is the only mediator in this model. However, given that motivation implies moving *toward* an academic task (Ryan and Deci, 2000) rather than *away* from an academic task like in procrastination, the model could have benefited from adding mediators that imply moving toward an academic task, such as resource management strategies (e.g., time and study environment, effort regulation; see Credé and Phillips, 2011). Therefore, adding other relevant mediators in this multilevel model could be an interesting expansion for future research. Another way to expand the model would be to not only measure personal antecedents (e.g., course motivation), but also contextual antecedents (e.g., course climate and course content) that could influence achievement and well-being via procrastination. Alternatively, researchers could simply examine whether the mediating paths found in this study could also be found with other samples, like high school students or graduate students. By having a better understanding of this multilevel model across various samples, it would be easier to propose and tailor future interventions to reduce procrastination both at a student level and at a course level.

## Conclusion

In this multilevel study, we found that some students procrastinate more than others (i.e., between-person level) and that students procrastinate more in some courses than in others (i.e., within-person level). In both cases, procrastination was significantly related to lower academic achievement and well-being. However, the antecedents of procrastination were not the same at each level of analysis. This finding suggests that applied psychologists attempting to reduce procrastination would benefit from determining the level at which they want to intervene. If the goal is to reduce the general academic procrastination of a student (compared to other students), it seems preferable to reduce controlled motivation toward school in general. If the goal is to reduce procrastination in a given course (compared to one’s own average), it seems preferable to increase autonomous motivation toward that course specifically. Future research will be needed to determine the optimal format and content of potential intervention programs to reduce procrastination at each level. Overall, our study demonstrated that a new understanding of academic procrastination can be gained by examining it from a multilevel perspective.

## Data Availability Statement

The raw data supporting the conclusions of this article will be made available by the authors, without undue reservation.

## Ethics Statement

The studies involving human participants were reviewed and approved by the Office of Research Ethics and Integrity, University of Ottawa. The Research Ethics Board of the University of Ottawa determined that minor participants (university students under the age of 18) participating in the integrated system for participation in research were not required to provide a written informed consent from a legal guardian/next of kin to participate in this study. Written informed consent from the participants’ legal guardian/next of kin was not required to participate in this study in accordance with the national legislation and the institutional requirements. The participants provided their written informed consent to participate in this study.

## Author Contributions

KK designed the study, collected the data, conducted the analyses, and wrote the manuscript. BS provided feedback on each version of the manuscript. PG helped with designing the study, conducting the analyses, and providing feedback on the manuscript. All authors contributed to the article and approved the submitted version.

## Conflict of Interest

The authors declare that the research was conducted in the absence of any commercial or financial relationships that could be construed as a potential conflict of interest.

## Publisher’s Note

All claims expressed in this article are solely those of the authors and do not necessarily represent those of their affiliated organizations, or those of the publisher, the editors and the reviewers. Any product that may be evaluated in this article, or claim that may be made by its manufacturer, is not guaranteed or endorsed by the publisher.

## Footnotes

1 Parameter estimates with fixed vs. random effects were compared using traditional multilevel modeling. The first model examined the relations from the two types of motivation to procrastination. The second model examined the relations from the two types of motivation and procrastination to the three outcomes. All the compared parameter estimates were very similar. Only the association between controlled motivation and grades was noticeably different in a model with fixed effects (*B* = −0.248, 95% CI = −0.469, −0.027, *p* = 0.028) vs. random effects (*B* = −0.202, 95% CI = −0.425, 0.022, *p* = 0.077). Although the statistical significance of this association changes from one model to another, the effect sizes are comparable. Furthermore, the association between controlled motivation and grades is not a central link in our mediation model and we have no evidence that this small change in effect size affects the rest of the model in significant ways. Overall, we believe that these sensitivity analyses suggest that our final results are most likely not affected in important ways by the absence of random effects in our MSEM model.

2
http://www.thestatisticalmind.com/calculators/SBChiSquareDifferenceTest.htm
